# Assessment of Prevalence of Malnutrition and Its Associated Factors among AIDS Patients from Asella, Oromia, Ethiopia

**DOI:** 10.1155/2020/7360190

**Published:** 2020-12-07

**Authors:** Teferi Teklu, Nitin Mahendra Chauhan, Firaol Lemessa, Getu Teshome

**Affiliations:** ^1^Department of Public Health, College of Health Sciences, Arsi University, Arsi, Ethiopia; ^2^Department of Biology, College of Natural and Computational Sciences, Dilla University, 419, Dilla, SNNPR, Ethiopia

## Abstract

Sub-Saharan Africa remains to be the most heavily affected region by malnutrition, accounting for 23.8% share of the global burden. Undernutrition weakens the immune system, increases the susceptibility to infections, and may worsen the impact on various kinds of diseases. Our aim was to assess undernutrition and its associated factors among AIDS-infected adult patients from Asella, Oromia Region, Ethiopia. An institutional-based cross-sectional study design was employed from June to July 2018. A total number of 519 patients were selected for the proposed work. Data was entered into EpiData, checked, coded, and analyzed using SPSS version 21 software. Descriptive statistics were used to assess the prevalence of undernutrition among patients. Bivariate and multivariate regressions were used to determine the relationship between undernutrition and its associated factors among the study participants. The results of our study showed that the overall prevalence of undernutrition was 18.3%; out of which 12.7% were mildly and 5.6% were moderately to severely undernourished, respectively. Monthly income (AOR: 3.589, 95% CI (1.469-8.768)), whole grain feeding (AOR: 2.979, 95% CI (1.252-7.088)), opportunistic infections in the last six months (AOR: 3.683, 95% CI (3.075-4.411)), clinical stage (AOR: 2.998, 95% CI (1.269-7.083)), and insufficient quality of food (AOR: 3.149, 95% CI (1.339-7.406)) were found to be significantly associated with undernutrition in this study. Therefore, HIV treatment facility should be supported with nutritional assessment, supplementation, counseling, care, and support to patients that may possibly alleviate this predicament.

## 1. Introduction

Human immunodeficiency virus (HIV) epidemic remains one of the major public health challenges globally. In 2018, 37.9 million peoples were living with HIV and 1.7 million with new HIV infections have been reported worldwide [1, 2]. More than 800 million peoples worldwide are chronically undernourished of which 200 million are living in Sub-Saharan Africa (SSA), and greater than 33 million are living with HIV infection [3, 4]. An estimated 0.8% of adolescents aged between 15 and 49 years worldwide are living with HIV currently. The burden of the epidemic continues to differ considerably between countries and regions. Sub-Saharan Africa remains most severely affected, with an early 1 in every 20 adults living with HIV and accounting for 71% of the people living with HIV globally [5].

The first evidence of HIV epidemic in Ethiopia was detected in 1984. Ethiopia was labeled to have the biggest epidemic with 1.5% of HIV prevalence adult peoples aged in the range 15 to 49 years, among 5 SSA [6]. Based on the 2014 estimate, 367,000 patients are taking antiretroviral therapy (ART). However, the need for ART is 542,121 for adults and 178,500 for children under 15 years of age for the year 2014 [7].

Food and nutrition are the basic needs for health, growth, and development, but in Africa, it has been a long-standing challenge to provide sufficient food and nutrition, which is also exacerbated by the human immunodeficiency virus/acquired immunodeficiency syndrome (HIV/AIDS) pandemic. Food is one of the most important needs of people with HIV/AIDS [8]. In resource-limited settings, many people living with HIV/AIDS lack access to sufficient quantities of nutritional foods, which poses additional challenges to the success of highly active antiretroviral therapy (HAART) [9, 10].

Nutrition and HIV are strongly related to each other since any immune impairment as a result of HIV/AIDS leads to malnutrition, and malnutrition leads to immune impairment, worsens the effect of HIV, and contributes to more rapid progression to AIDS. Asymptomatic HIV-positive individuals require 10% more energy, and symptomatic HIV-positive individuals require 20-30% more energy than HIV-negative individuals of the same age, sex, and physical activity level. Low food intake combined with increased energy demands are the major factors in HIV-related weight loss and wasting [11].

Studies have shown that sociodemographic factors also have an effect on nutritional status. Meta-analysis of 11 Sub-Saharan countries including Ethiopia identified that the magnitude of malnutrition among HIV patients varies by wealth status, educational attainment, occupation, and type of residence [12]. The Ghanaian study identified that nutritional status was significantly associated with marital status, income per month, educational level, and large family size [13]. Similarly, another study from Gondar identified that marital status, income, duration of treatment, eating problem, and current status of the patient are significantly associated with undernutrition [14].

Though HIV/AIDS is known to aggravate the occurrence of undernutrition, there is no study conducted to evaluate the effect in the study area. Therefore, the aim of our study is to assess the prevalence of malnutrition and factors associated with HIV/AIDS peoples from Asella, Oromia Region, Ethiopia.

## 2. Materials and Methods

### 2.1. Study Area and Study Design

A facility-based cross-sectional study was conducted among HIV adult patients receiving HAART in Assela Town, Oromia, Ethiopia. The town is located in Arsi Zone, Oromia, Ethiopia, and is situated at a distance of 175 kilometers to the southeast of Addis Ababa, the capital city of Ethiopia [15]. According to the Asella Town Administration Office report (2018/2019), the current population of Asella Town is estimated to be 110,433 with proportion of 55,990 males and 54,443 females [16]. The study was conducted from June to July 2018.

### 2.2. Sampling Procedure

The sample size, i.e., 519, was determined by using the formula for single population proportion by considering the following assumptions: a 95% confidence level, 5% margin of error, and *p* = 31% from the estimated proportion of malnutrition and associated factors among adult individuals on HAART in Asella [16]. Followed by 10% nonresponse rate was added. Finally, the correction formula was used as source of population less than 10,000 and was modified to 3984.

All public health facilities in Asella Town providing ART service were included in the study. From the total of 4324 people living with HIV (PLHIV), 3984 (3356 from Asella Hospital and 628 Asella Health Center) were adults above 15 years of age. Therefore, in order to select 519 participants from 3984 PLHIV proportionately, 437 from Asella Hospital and 82 from Asella Health Center were selected. Based on eligibility criteria people living with HIV on HAART, a systematic random sampling was used to select samples (clinical record of patients on log book). Sampling interval *k*^th^ was determined by dividing the total patients actively on ART in each of the health facilities by the required sample size (*K*^th^ = *N*/*n* = 3984/519 = 8). From the total PLHIV on HAART in sample, the first clinical record was selected by a simple random sampling and every 8^th^ client was selected for gathering information until the required sample was obtained. Finally 519 adult PLHIV on HAART were selected for the proposed study.

### 2.3. Data Collection Process and Tool

Weights of participants were taken by using a standard beam balance, and the scale was adjusted to zero before and after each measurement. Participant's weight was measured after removing heavy clothes and was recorded to the nearest 0.1 kg. Height measurement of participants was taken using the standard measuring scale. Body mass index (BMI) was calculated as weight in kilograms divided by the square of height in meters (kg/m^2^). For the initial analysis, BMI was stratified according to the WHO criteria: <17 kg/m^2^ (moderate to severe malnutrition), 17 to <18.5 kg/m^2^ (mild malnutrition), >18.5 to 25 kg/m^2^ (normal nutrition), and >25 kg/m^2^ (overweight and obese) [17].

Patient's medical chart was reviewed for extraction of AIDS clinical stage data and history of previous opportunistic infections (OIs) in the last 6 months. Blood samples were drawn from subjects as part of routine monthly ART followed by investigation to measure CD4 cell count. The proposed study used CD4 cell count to classify the patients into three categories according to WHO criteria: <200 cells/mm^3^ (severe), 200–499 cells/mm^3^ (moderate), and ≥500 cells/mm^3^ (mild).

Using the FAO Nutrition and Consumer Protection Division recommended questionnaire for data collection on individual dietary diversity score (IDDS), a record of 24-hour recall of all food items eaten by the respondents was taken and classified into 12 different food groups [18]. Using FANTA (Food and Nutrition Technical Assistance), the Household Food Insecurity Access Scale (HFIAS) occurrence questions related to three different domains of food insecurity were determined.

## 3. Results

### 3.1. Sociodemographic Characteristics of the Respondents

More than half of the respondents (280) (53.90%) belong to the age groups of 30-45 years, while 83 (16%) were between 18 and 29 years. The mean age was 41.0231 ± SD 11.5055. More than six out of ten, 333 (64.20%) and 317 (61.10%), of the respondents belong to Orthodox Christians and Oromo Ethnic group, respectively, while 6 (1.20%) and 4 (0.80%) of the participants represent Catholic and Tigre, respectively. In this study, 184 (35.80%) were married. Regarding the educational status of respondents, 183 (35.30%) were only able to attend elementary school; on the other hand, 36 (6.90%) had studied at the college level and above. The employment status of respondents in this study was 164 (31.60%) farmers and only 10 (2%) of PLHIV were unemployed (Table 1).

### 3.2. Clinical Profiles and ART Status of Study Participants

The HIV status of patients showed that almost three out of ten, 152 (29.35%), seen HIV-related symptom two weeks prior to the data collection period. Also, 37 (26.4%) were not able to feed properly; majority of them, 87 (63.5%), had loss of appetite. In this study, 483 (93.10%) were at WHO clinical stage I, 123 (23.70%) were diagnosed with opportunistic infection within the last six months, and majority, 282 (54.30%), had moderate CD4 category with a CD4 count of 200-499 cells/mm^3^. Most of the patients, 335 (64.5%), have taken HAART for more than three years; among them, 9 (1.7%) had ART drug side effect (Table 2).

### 3.3. Food- and Lifestyle-Related Characteristics

The study showed that 512 (98.7%) adult patients had counseled about their feeding style after knowing their HIV status. About 461 (88.8%) of them have received general feeding counseling. The most common substances ever and currently used in the study area reported by participants were khat/shisha (131 (25.2%) and 20 (3.9%), respectively). Moreover, among all patients, 17 (3.3%) have taken hard drugs at least once in their lifetime (Table 3).

### 3.4. Dietary Diversity Scores (DDS)

The most commonly eaten foods within the past 24 hours before data collection were cereals (394/519, 75.9%) and other foods consumed by the respondents which include condiments like coffee/tea (462/519, 89%), legumes, nuts, and seeds (431/519, 83%), and roots or tubers (337/519, 64.9%). The food groups eaten by less than 50% of the participants were fish and other seafoods, milk and milk products, fruits, vegetables, meat and meat products, eggs, and oil fat or butter (Table 4).

**Figure 1:**
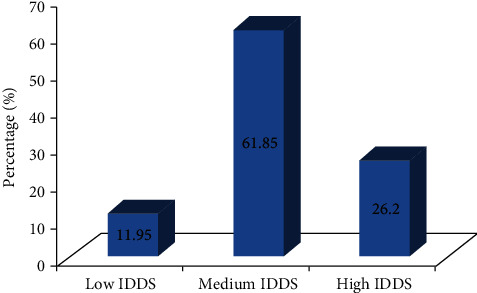
Individual dietary diversity score of the study participants in Asella Town, Southeast Ethiopia.

FAO Nutrition and Consumer Protection Division recommended questionnaire for data collection on IDDS which revealed that 61.85% of respondents had medium IDDS, 26.2% had high IDDS, and 11.95% had low IDDS (Figure 1).

**Figure 2:**
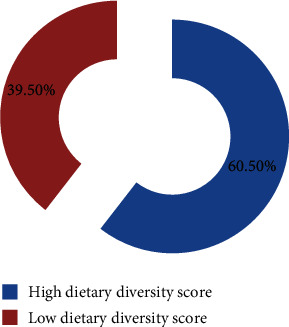
Dichotomous category of the total individual dietary diversity scores.

According to the dichotomous category of the total individual food scores, 205 (39.5%) participants had low dietary diversity (≤4 food groups) and 314 (60.5%) had high dietary diversity (≥5 food groups) per 24 hours before data collection (Figure 2).

### 3.5. Household Food Insecurity Access Scale (HFIAS)

Based on HFIAS indicator categorization, 30.2% of households had secured food, 34.8% of patients were reported to have mild Food Insecurity Access, 25.6% of respondents have moderate Food Insecurity Access, and 9.2% of participants were found to be severely food insecure (Table 5).

### 3.6. Prevalence of Undernutrition

**Figure 3:**
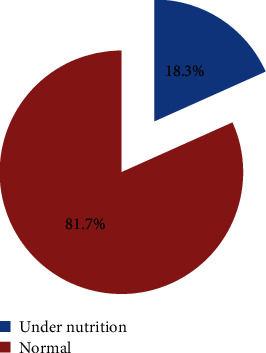
Prevalence of undernutrition among PLHIV in Asella Town, Southeast Ethiopia.

Among 519 PLHIV participated in this study, 95 (18.3%) respondents had BMI < 18.5 kg/m^2^ with the corresponding 95% confidence interval of 14.9665-21.6423; among these, 61 (64.20%) were females and half of 48 (50.5%) were between 30 and 45 years old. From the total of 95 (18.3%) undernourished PLHIV, 66 (12.70%) were mildly malnourished and 29 (5.60%) were moderately to severely malnourished. Therefore, the overall undernutrition in this study was found to be 18.3% (Figure 3).

### 3.7. Factors Associated with Undernutrition

The multivariable logistic regression analysis was used by taking all the factors into account simultaneously, and five of the most contributing factors remained to be significantly and independently associated with undernutrition which includes monthly income, whole grain feeding, insufficient quality feeding, opportunistic infection, and clinical stages. Monthly income had showed a statistically significant association with outcome variable. Those participants whose average monthly income was below 13.5 USD were 3.589 times more likely to be undernourished compared to those whose income was 54 USD and above (AOR: 3.589, 95% CI (1.469-8.768)). It was found that daily whole grain food intake of ART patient was found to be one of the determinants of undernutrition; patients who did not feed grain and grain products in a day were almost three times more likely to be undernourished than patients who eat grain and grain products (AOR: 2.979, 95% CI (1.252-7.088)) (Table 6).

Illness of a patient within WHO clinical stage was one of the significant risk factors of undernutrition in the study area. Those patients with the illness at WHO clinical stage II were approximately three times more likely to be undernourished than WHO clinical stage I ART patients (AOR: 2.998, 95% CI (1.269-7.083)). Patients who had a current or past six-month history of opportunistic infections were 3.683 more likely to be undernourished than those who were not infected with OIs (AOR: 3.683, 95% CI (3.075-4.411)) (Table 6).

Yet again, insufficient quality of feeding among patents became one of the risk factors which significantly associated with undernutrition; those patients who feed insufficient quality of food for 3-10 times per month were more than three times more likely to be undernourished than those who feed once or twice per month (AOR: 3.149, 95% CI (1.339-7.406)) (Table 7).

## 4. Discussion

The proposed institutional-based cross-sectional study attempts to assess the prevalence of undernutrition and its associated factors among adult PLHIV and revealed that the overall prevalence of undernutrition was 18.3%. Magnitude of undernutrition in PLHIV is important because it may predict disease progression and higher risk of morbidity and mortality. The presence of undernutrition is a predictor of worse outcome in HIV-infected individuals [19].

The overall prevalence of undernutrition in this study is consistent with the studies done in Nepal (19.9%) [20] and Tanzania (18.4%) [21]. The proportion of undernutrition of adult PLHIV in this study is higher than the studies done in Dilla, Ethiopia (12.3%) [22] and Kenya (9.8%) [23]. However, the prevalence of undernutrition was lowest compared to the study reports from other parts of Ethiopia, i.e., Humera (42.3%) [24] and Dembia (23.2%) [25], and other developing countries, such as Botswana (28.5%) [26] and Nigeria (43.3%) [27]. Observed discrepancy could be attributed to the clinical stage of the study participants, where majority (93.1%) of them are found at the clinical stage I in the current study. This different result of undernutrition among different parts of the country shows that there is existence of different socioeconomic and other factors that predispose the community to the problem, probably different feeding styles of different ethnic groups in the country. As such, the difference of undernutrition may reflect due to population difference, sample, and year of study.

Undernutrition could occur in different forms and degrees. When we consider the degree of undernutrition, it varies in different circumstance. In this study, from the total undernutrition, 12.7% were mild and 5.6% were moderate to severe undernutrition. In other studies, the proportion of the degree of undernutrition was 20.3% mildly, 22% moderately to severely and 9% mildly, 3.5% moderately to severely malnourished [24–27]. From the above descriptive results, we looked at differences in the distribution of degree of undernutrition and what is clearly seen is HIV/AIDS-related undernutrition is the major problem among AIDS patients.

Descriptively, from the total participants on ART, females accounted about 310 (59.7%), and from whom, 66 (69.4%) were undernourished. Males accounted 40.3% of which 29 (30.6%) were undernourished. From the total malnourished, the proportion of malnutrition was much higher in females who were on ART care (69.4%) when compared to males (30.6%). The proportion of women undernourished in this study is higher than the study conducted in Tigray Humera Hospital, i.e., 42.3% [24], and Felegehiwot Referral Hospital, Bahir Dar (52%) [28]. Probably, it might be due to different sociocultural, residence, and dietary diversity.

Results of our study showed that monthly income had showed statistically significant association with outcome variable. Participants with average monthly income below 13.5 USD were 3.589 times more likely to be undernourished when compared to those whose income was 54 USD and above (AOR: 3.589, 95% CI (1.469-8.768)). As evidenced by other study, it revealed that there was more than 50% decrease in an average monthly household income among HIV-affected households than non-HIV-affected households because of HIV-related mortality coupled with high medical expense [29]. This might be explained as having good economic status creates better opportunity to secure food availability and to purchase varieties of nutritional foods. As a result, dietary habit or food consumption pattern of HIV-positive individuals with poor economic status may largely base on low-cost, least nutritious, and monotonous food groups.

The current study showed that WHO clinical stage is one of the significant associated factors with undernutrition; patients who were in WHO clinical stage I are less likely to develop undernutrition than a patient in stage II. Those patients with the illness at WHO clinical stage II were approximately three times more likely to be undernourished than WHO clinical stage I ART patients (AOR: 2.998, 95% CI (1.269-7.083)) in this study. This finding is not supported by studies reported from Dilla Referral Hospital, Dilla, Ethiopia [18], where WHO clinical stages III and IV have statistically significant association with undernutrition. Other study also revealed that individuals at all stages of HIV disease are at risk of nutritional deficiency, but clinical stages show the severity of the disease from primary HIV infection to advanced stages of HIV or AIDS [30]. Undernutrition is usually encountered at the advanced phase of the HIV infection, and anthropometric measurements are lower in symptomatic HIV/AIDS patients classified by WHO stages [31]. This discrepancy might be due to the clinical stage of the study participants, where majority (93.1%) of them are found at the clinical stage one in the current study.

Regarding OIs, individuals who were diagnosed with two or more OIs during the past six months were 3.7 times more likely to be undernourished than not diagnosed with OI (AOR: 3.683, 95% CI (3.075-4.411)). This finding was consistent with other studies in southern Ethiopia [18] and Kenya [32]; number of previous OIs significantly associated with undernutrition. Evidences also stated that the HIV-induced immune impairment and opportunistic infections can worsen nutritional status and OIs placing PLHIV at a high risk of developing malnutrition [33].

We also assessed dietary diversity score of PLHIV which was measured by the total number of food groups that PLHIV (any member of the household in which the PLHIV are on ART) consumed during 24 hours prior to the study. In this study, 205 (39.5%) of the study participants had low dietary diversity, which is lower than the reports from a study in Metema Hospital (58.8%) [34] and in Jimma University Specialized Hospital in Ethiopia (55.8%) [35]. This shows that HIV-positive adults attending in the present study area had better dietary intake compared to what is revealed in the previous studies, with dietary diversity (the number of foods consumed across and within food groups over a reference period). This could be related to poor dietary habit of HIV-positive adults. The food types frequently consumed during 24 hours at the study period by the study participants were cereals (75.9%), condiments/coffee/tea (89%), legumes, nuts, and seeds (83%), and roots or tubers (64.9%). Fish (2.3%) and eggs (9.6%) were the least number of food groups consumed by PLHIV study participants. These differences could be attributed to seasonality, geographical differences, and absence of this food source in this study area.

Household access to food is a key indicator for predicting undernutrition. Majority (362) (69.8%) of the study participants were food insecure. The result of this study is consistent with study conducted in Hossana, Ethiopia (68.5%) [36]. However, this finding is higher than the studies conducted in Jimma Referral Hospital, Ethiopia (63.0%) [35] and Humera, Ethiopia (40.4%) [24] of PLHIV which were food insecure, while it is lower than the one conducted in Fitche, Ethiopia (87%) [37] and Dembia, Ethiopia (81.6%) [25] of the households which were food insecure. This might be due to the variation in the socioeconomic status of study areas and also could be attributed to seasonality, geographical differences, study population size, and study design. However, the cross-sectional design was not able to establish temporality between the determinants and the outcome variable. Therefore, continuous nutrition therapy and early treatment of opportunistic infection at the facility level for target clients improve household income through creating employment opportunities and engaging PLHIV in different income generating activities which could possibly alleviate this predicament.

## 5. Conclusion

In this study, the prevalence of undernutrition among HIV-positive adults was 18.3%. Furthermore, monthly income, whole grain feeding, OIs in the last 6 months, WHO clinical stage, and insufficient quality of food were found significantly associated with undernutrition from this study. About 39.5% of the study participants had low dietary diversity, and majority (69.8%) of the study participants were food insecure. Therefore, HIV treatment facility should be supported with nutritional assessment, supplementation, counseling, care, and support to patients. A comprehensive nutritional assessment and support should be provided for all patients on follow-up care. Moreover, community support to patients should be strengthened, as social determinants of health may also interact with effectiveness of treatments.

## Figures and Tables

**Table 1:** Sociodemographic characteristics of PLHIV in Asella Town, Southeast Ethiopia.

Variables (*n* = 519)	Frequency	Percentage (%)
Age		
18-29	83	16
30-45	280	53.9
>45	156	30.1
Sex		
Male	209	40.3
Female	310	59.7
Ethnicity		
Oromo	317	61.1
Amhara	168	32.4
Gurage	24	4.6
Tigray	4	.8
Others (Sidama, Hadiya, and Wolaita)	6	1.2
Religion		
Orthodox	333	64.2
Muslim	145	27.9
Protestant	35	6.7
Catholic	6	1.2
Educational status		
Unable to read and write	73	14.1
Read/write but no formal education	68	13.1
Elementary school	183	35.3
Secondary school	112	21.6
Grade 12 complete	47	9.1
College and above	36	6.9
Occupation		
Farmer	164	31.6
Full-time housewife	54	10.4
Housewife with occasional small-scale trade	69	13.3
Merchant	72	13.9
Governmental employee	37	7.1
Nongovernmental employee	19	3.7
Student	31	6.0
Day laborer	63	12.1
Jobless	10	2
Marital status		
Single	42	8.1
Married	184	35.5
Divorced	144	27.7
Widowed	111	21.4
Live-in cohabiting	38	7.3
Monthly income (USD)		
<13.5	131	25.2
13.5-27	167	32.2
27-54	130	25.1
54-135	91	17.5
Head of household		
Male	321	61.8
Female	198	38.2
Family size		
<3	271	52.2
3-6	229	44.1
≥7	19	3.7

**Table 2:** Clinical profiles and ART status of the study participants in Asella Town, Southeast Ethiopia.

Variables (*n* = 519)	Frequency	Percentage (%)
HIV-related symptoms 2 weeks prior to survey		
Yes	152	29.3
No	367	70.7
Eating problem		
Yes	137	26.4
No	382	73.6
Problems		
Swallowing difficulty	23	16.7
Loss of appetite	87	63.5
Vomiting	32	23.3
Opportunistic infections in the past 6 months		
None	396	76.3
1	111	21.4
2+	12	2.3
Clinical stage		
Stage I	483	93.1
Stage II	22	4.2
Stage III	14	2.7
CD4 count		
<200	40	7.7
200-499	282	54.3
≥500	197	38.0
Duration of HAART		
6 months-3 years	184	35.5
≥3 years	335	64.5
Side effect of HAART		
Yes	9	1.7
No	510	98.3

**Table 3:** Food- and lifestyle-related characteristics of the study participants in Asella Town, Southeast Ethiopia.

Variables (*n* = 519)	Frequency	Percentage (%)
Ever had nutritional counseling		
Yes	512	98.7
No	7	1.3
Type of counseling		
Drugs	408	78.6
Infection/illness	31	6
General feeding	461	88.8
Ever smoked		
Yes	80	15.4
No	439	84.6
Currently smoking		
Yes	9	1.7
No	510	98.3
Ever-drunk alcohol		
Yes	199	38.3
No	320	61.7
Currently drinking		
Yes	10	1.9
No	509	98.1
Ever-used khat/shisha		
Yes	131	25.2
No	388	74.8
Currently used khat/shisha		
Yes	20	3.9
No	499	96.1
Ever had hard drugs (cocaine, morphine, etc.)		
Yes	17	3.3
No	502	96.7

**Table 4:** Food variety consumption characteristics of the study participants in Asella Town, Southeast Ethiopia.

Variables (*n* = 519)	Frequency	Percentage (%)
Whole grain feeding		
Yes	394	75.9
No	125	24.1
Feeding foods made from roots or tubers		
Yes	337	64.9
No	182	35.1
Vegetables		
Yes	177	34.1
No	342	65.9
Fruits		
Yes	62	11.9
No	457	88.1
Meat and meat products		
Yes	91	17.5
No	428	82.5
Any eggs		
Yes	50	9.6
No	469	90.4
Any fresh or dried fish or shellfish		
Yes	12	2.3
No	507	97.7
Any foods made from beans, peas, lentil, or nuts		
Yes	431	83.0
No	88	17.0
Milk or other milk products		
Yes	115	22.2
No	404	77.8
Foods made with oil, fat, or butter		
Yes	225	43.4
No	294	56.6
Sugar or honey		
Yes	143	27.6
No	376	72.4
Other foods, such as condiments, coffee, or tea		
Yes	462	89.0
No	57	11.0

**Table 5:** Household Food Insecurity Access Scale (HFIAS) of study participants in Asella Town, Southeast Ethiopia.

HFIAS questions (*n* = 519)	Yes (%)	No (%)
Worry about food	231 (44.5)	288 (55.5)
Unable to eat preferred foods	295 (56.8)	224 (43.2)
Eat a limited variety of foods	303 (58.4)	216 (41.6)
Eat foods that you really did not want to eat	195 (37.6)	324 (62.4)
Eat a smaller meal	152 (29.3)	367 (70.7)
Eat fewer meals in a day	120 (23.1)	399 (76.9)
No food to eat of any kind in the household	28 (5.4)	491 (94.6)
Go to sleep at night hungry	16 (3.1)	503 (96.9)
Go a whole day and night without eating anything	2 (0.4)	517 (99.6)

**Table 6:** Dose response of Household Food Insecurity Access Scale (HFIAS) of study participants in Asella Town, Southeast Ethiopia.

HFIAS questions	Frequency (*n*)	Rarely (%)	Sometimes (%)	Often (%)
Worry about food	231	157 (70)	57 (24.6)	17 (7.4)
Unable to eat preferred foods	295	181 (61.4)	104 (35.2)	10 (3.4)
Eat a limited variety of foods	303	149 (49.2)	133 (43.9)	21 (6.9)
Eat foods that you really did not want to eat	195	112 (57.5)	58 (29.7)	25 (12.8)
Eat a smaller meal	152	103 (67.7)	44 (30)	5 (3.3)
Eat fewer meals in a day	120	81 (67.5)	38 (31.6)	1 (0.9)
No food to eat of any kind in the household	28	17 (60.7)	11 (39.3)	0
Go to sleep at night hungry	16	14 (87.5)	2 (12.5)	0
Go a whole day and night without eating anything	2	2 (100)	0	0

**Table 7:** Bivariate and multiple logistic regression analysis of factors associated with undernutrition among people living with HIV/AIDS in Asella Town, Southeast Ethiopia.

Variables (*n* = 519)	Undernutrition status		COR (95% CI)	AOR (95% CI)
	Undernourished	Normal		
*Monthly income*				

<500	34	97	**4.206 (1.772-9.983)** ^∗^	**3.589 (1.469-8.768)** ^∗∗^
500-1000	36	131	**3.298 (1.403-7.752)** ^∗^	**3.221 (1.333-7.736)** ^∗∗^
1000-2000	18	112	1.929 (0.770-4.828)	2.201 (0.856-5.663)
2000-5000	7	84	1	1

*Duration of HAART*				

6 months-3years	25	159	**0.595 (0.362-0.979)** ^∗^	0.794 (0.329-1.919)
≥3 years	70	265	1	1

*Whole grain feeding*				

Yes	61	333	1	1
No	34	91	**2.040 (1.263-3.294)** ^∗^	**2.979 (1.252-7.088)** ^∗∗^

*Opportunistic infection in the last 6 months*				

None	53	330	1	**1**
1	33	85	**2.417 (1.417-3.968)** ^∗^	2.146 (0.867-5.315)
2+	9	9	**6.226 (2.364-16.399)** ^∗^	**3.683 (3.075-4.411)** ^∗∗^

*Alcohol drinking*				

Yes	38	161	1.089 (0.691-1.716)	1.386 (0.579-3.314)
No	57	263	1	1

*Clinical stage*				

Stage I	79	397	**1**	1
Stage II	12	17	**3.574 (1.630-7.718)** ^∗^	**2.998 (1.269-7.083)** ^∗∗^
Stage III	4	10	2.010 (0.615-6.571)	1.268 (0.293-5.485)

*Insufficient quality*				

Rarely (one or twice/month)	17	95	1	1
Sometimes (3-10 times/month)	19	39	**2.772 (1.282-5.781)** ^∗^	**3.149 (1.339-7.406)** ^∗∗^
Often (> ten times/month)	1	24	0.233 (0.030-1.838)	0.317 (0.037-2.719)

^∗^Association on bivariate analysis. ^∗∗^Statistically significant.

## Data Availability

The data used to support the findings of this study are available from the corresponding author upon request.

## Abbreviations

AIDS: Acquired immune deficiency syndrome
AOR: Adjusted odds ratio
ART: Antiretroviral therapy
BMI: Body mass index
CD4: T lymphocyte bearing CD4 receptor
CI: Confidence interval
DHS: Demographic and health survey
FAO: Food and Agriculture Organization
HAART: Highly active antiretroviral therapy
HFIAS: Household Food Insecurity Access Scale
HIV: Human immunodeficiency virus
IDDS: Individual dietary diversity score
OIs: Opportunistic infections
OR: Odds ratio
PLHIV: People living with HIV/AIDS
SSA: Sub-Saharan Africa
WHO: World Health Organization.
